# Smart sanitization on a budget: revolutionizing public transport hygiene post-COVID

**DOI:** 10.3389/fpubh.2024.1437499

**Published:** 2024-07-19

**Authors:** J. Dinesh Peter, D. Raveena Judie Dolly, D. J. Jagannath, S. Allen Livingston

**Affiliations:** ^1^Division of Artificial Intelligence and Machine Learning, Karunya Institute of Technology and Sciences, Coimbatore, India; ^2^Division of Electronics & Communication Engineering, Karunya Institute of Technology and Sciences, Coimbatore, India; ^3^Division of Mechanical Engineering, Karunya Institute of Technology and Sciences, Coimbatore, India

**Keywords:** public transport hygiene, sanitization, post-COVID, hygiene, public transport systems, smart sanitization

## Introduction

In light of the widespread transmission of COVID-19, a novel smart sanitizing device is proposed to curb the rapid spread of the virus. The potential health hazard posed by hand contact on the handrails of public transport vehicles underscores the urgency to efficiently ensure clean handrails. The innovative system incorporates spring-powered wheels, serving as an adjustable clamping mechanism, alongside a polymer brush and a container for a disinfectant solution. This device operates automatically on both horizontal and vertical handrails through a clockwork mechanism, offering advanced hygiene protection by continuously and automatically disinfecting surfaces from coronaviruses.

Handrails, frequently touched and potential breeding grounds for bacteria and viruses, pose significant health risks in heavily frequented areas like public transportation, hospitals, and educational institutions. To address this concern, we introduce a handrail sanitizing device utilizing a liquid sanitizer, primarily composed of Isopropyl Alcohol, known for its efficacy in eradicating bacteria and viruses. The device is designed for easy installation and operation, ensuring adaptability to various handrail types. This summary provides an overview of the Low-Cost Handrail Sanitizing Device project, outlining the addressed problem, its features, advantages, and potential applications. The ongoing global COVID-19 pandemic has underscored the critical importance of implementing effective and accessible hygiene measures in public spaces to mitigate the spread of the virus. Public transport systems, serving as vital conduits for daily commuting, present a unique challenge due to the high frequency of human interaction and the potential for surface contamination. Among commonly touched surfaces, handrails in public transport vehicles have been identified as potential vectors for the transmission of infectious agents. In response to this pressing need, this research focuses on the design and development of a low-cost smart sanitizing device specifically tailored for handrails in public transport.

**Figure 1:**
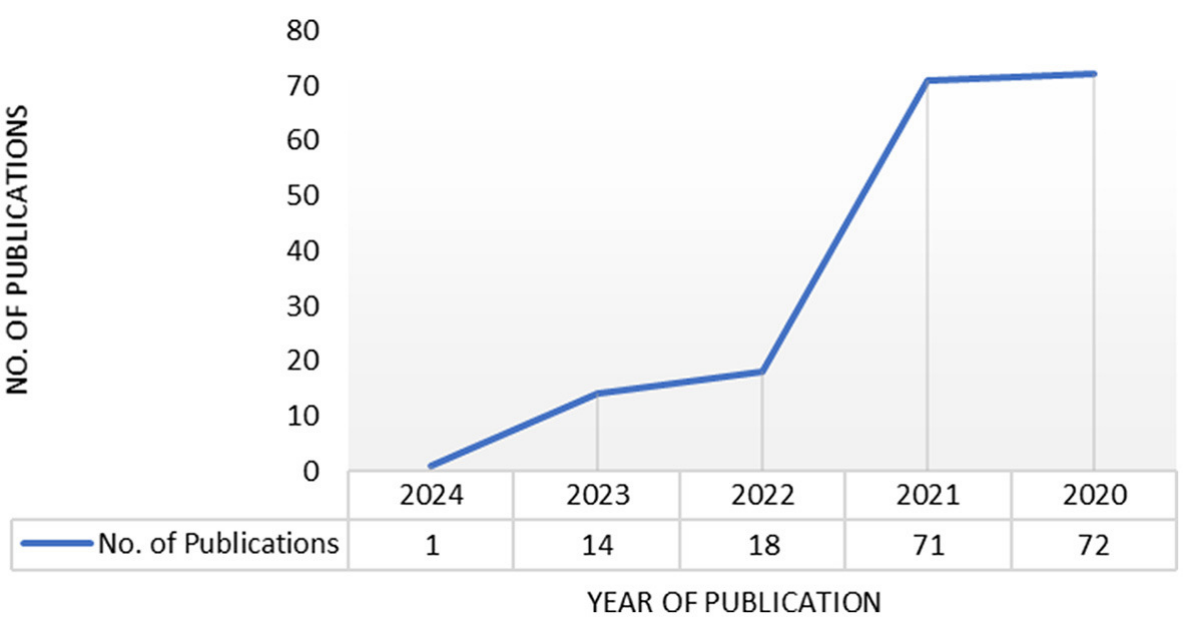
Research publications for sanitizing device to control COVID.

In exploring the research landscape related to sanitizing devices for controlling COVID-19, a notable trend emerges when considering the publication years. The search query conducted on PubMed indicates a substantial body of literature, with a comprehensive distribution across the years. The data reveals a consistent and growing interest in this field, with 72 publications in 2020, 71 in 2021, 18 in 2022, 14 in 2023, and 1 in 2024. This trend suggests a sustained focus on developing and understanding sanitizing devices, reflecting the dynamic nature of research efforts to address the ongoing challenges posed by the COVID-19 pandemic. The consistent publication output in 2020 and 2021 may signify the immediate response of the scientific community to the urgent need for effective sanitization solutions. The subsequent years, 2022 and 2023, continue to demonstrate ongoing research, indicating a sustained commitment to refining and advancing these technologies. The single publication in 2024 suggests that research in this domain remains an active area of investigation as shown in Figure 1, albeit with a potentially evolving focus or specialization as the pandemic and related technologies progress. Overall, this cumulative body of work reflects the persistent commitment of researchers to contribute innovative solutions in the ongoing fight against the spread of COVID. The current state of sanitization technologies in public transport include manual disinfection by cleaning staff using chemical sprays or wipes, automated systems such as UV-C light or misting systems, and the integration of antimicrobial materials in high-touch surfaces.

However, these existing technologies have several limitations:

1. **Manual processes**: Manual disinfection methods rely on human intervention, which can be inconsistent and labor-intensive. Cleaning schedules may vary, leading to potential lapses in sanitization efforts.
2. **Limited coverage**: In manual cleaning reaching all surfaces in the handrail is difficult to access and time consuming. So missed spots can cause infection for the passengers.
3. **Time consuming**: Manual disinfection processes can be time-consuming, leading to disruptions in service schedules and potentially impacting the efficiency of public transport systems.
4. **Chemical hazards**: Chemical disinfectants used in spraying systems may pose health risks to passengers and staff if not properly managed or if residues are not adequately removed.
5. **Sustainability concerns**: Misting sanitization methods may raise environmental concerns due to the use of chemicals or energy-intensive processes.

The proposed smart sanitizing device aims to provide an innovative and automated solution to address the hygiene concerns associated with handrails in public transport settings. By integrating advanced sensing technologies and efficient sanitization mechanisms, the device seeks to offer a practical and user-friendly solution for reducing the risk of viral transmission through contact surfaces. This research not only addresses the immediate challenges posed by the COVID-19 pandemic but also contributes to the broader discourse on enhancing public health measures in shared spaces, emphasizing the importance of technology-driven interventions in safeguarding public wellbeing. Through the development of this low-cost smart sanitizing device, we envision a scalable and adaptable solution that can be easily implemented in diverse public transport systems, offering a sustainable means to control the spread of COVID-19 and potentially other infectious diseases in the future.

## Materials and methods

The studies encompass a diverse array of materials and methods in addressing various facets of the COVID-19 pandemic. The investigation into nosocomial infections of COVID-19 by Du et al. (1) emphasizes the challenges confronted by healthcare professionals, shedding light on the unique issues encountered within healthcare settings. Amalou et al.'s (2) work introduces a novel approach with a disposable isolation device designed for CT scanning, aiming to minimize COVID-19 contamination and highlighting its potential impact on infection control in medical imaging procedures. Zamir et al.'s (3) research explores non-pharmaceutical interventions as effective measures for controlling COVID-19 spread, offering insights into alternative approaches. Ham's (4) study focuses on air purification, discussing challenges and concerns surrounding the use of air purifiers as a preventive measure against exposure to and spread of COVID-19. Zar et al.'s (5) investigation addresses the challenges specific to COVID-19 in pediatric cases, particularly in low- and middle-income countries, providing crucial insights into child health during the pandemic. Banerjee et al.'s (6) research delves into the psychological aspects influencing public behavior through an examination of prediction biases in exponential growth predictions related to COVID-19. The study by Zanardo et al. (7) concentrates on patient management within the radiology department, offering guidelines for effective healthcare practices during the pandemic. Pediatric dermatology perspectives on COVID-19 are provided by an article, contributing to a holistic understanding of the disease in children. Voirol et al.'s (8) work explores ways to mitigate the environmental and social impacts of the COVID-19 pandemic, suggesting avenues for sustainable practices. Verbeek et al.'s (9) study reviews the use of personal protective equipment (PPE) in preventing highly infectious diseases among healthcare staff, providing valuable insights for frontline workers' safety. Ubom et al.'s (10) research delves into socio-economic aspects, discussing the implications of prolonged lockdowns and COVID-19 vaccine costs in a low-middle income country. Tuñón-Molina et al.'s (11) article provides an overview of the status and future trends of protective face masks, exploring innovations and trends in mask development and usage for safeguarding against COVID-19. O'Donnell et al. (12) trace the historical evolution of medical uniforms, discussing their significance during the COVID-19 pandemic. Khalefa et al.'s (13) study focuses on safe practices beyond personal protective equipment in operating theaters during the COVID-19 pandemic. Doyon et al.'s (14) investigation explores the ultraviolet protection offered by masks during the COVID-19 pandemic. Maina et al.'s (15) study addresses challenges and opportunities in infection prevention and control in Kenyan public hospitals during the COVID-19 pandemic. Ham's (4) article reiterates the importance of air purification, emphasizing challenges and concerns related to preventing exposure to and spread of COVID-19 using air purifiers. Lastly, Dev et al.'s (16) research presents findings from a case-control study, highlighting risk factors and frequencies of COVID-19 among healthcare workers in an Indian tertiary care center, contributing valuable data for understanding and mitigating risks in healthcare settings.

## Methodology

### Design and prototyping

The design and development of the low-cost smart sanitizing device for handrails in public transport will be approached through a systematic and iterative process. Initially, a comprehensive review of existing sanitization technologies, materials, and sensor systems will be conducted to inform the conceptualization of the device. The design will prioritize cost-effectiveness, ease of integration into existing public transport infrastructure, and user-friendly operation. Computer-aided design (CAD) software will be employed to create detailed schematics and 3D models, allowing for visualization and refinement of the device's physical structure.

Following the design phase, prototyping will be a crucial step to validate the feasibility and functionality of the device. The prototype will incorporate the proposed sensing technologies, such as infrared or ultrasonic sensors for detecting handrail usage, and a sanitization mechanism, potentially utilizing a controlled release of a sanitizing agent. Testing under simulated conditions will assess the efficacy of the sanitization process and ensure that the device complies with safety standards. Feedback from these initial prototypes will guide iterative improvements in subsequent design iterations.

### Integration and field testing

Once the prototype has been refined through multiple iterations, the focus will shift to the integration of the smart sanitizing device into actual public transport environments. Collaborations with relevant stakeholders, including transport authorities and vehicle manufacturers, will be sought to facilitate seamless integration. Field testing will involve installing the devices on select public transport routes to gather real-world data on usability, durability, and the effectiveness of the sanitization process.

User acceptance and feedback will be actively sought during this phase to identify any practical challenges or improvements needed. Data analytics will play a crucial role in assessing the device's performance, including the frequency of usage, sanitization success rates, and overall impact on reducing surface contamination. The methodology will be adaptive, allowing for adjustments based on the insights gained from field testing, with the ultimate goal of delivering a reliable, cost-effective, and widely applicable smart sanitizing solution for handrails in public transport.

### Phases of the project methodology

**Figure 2:**
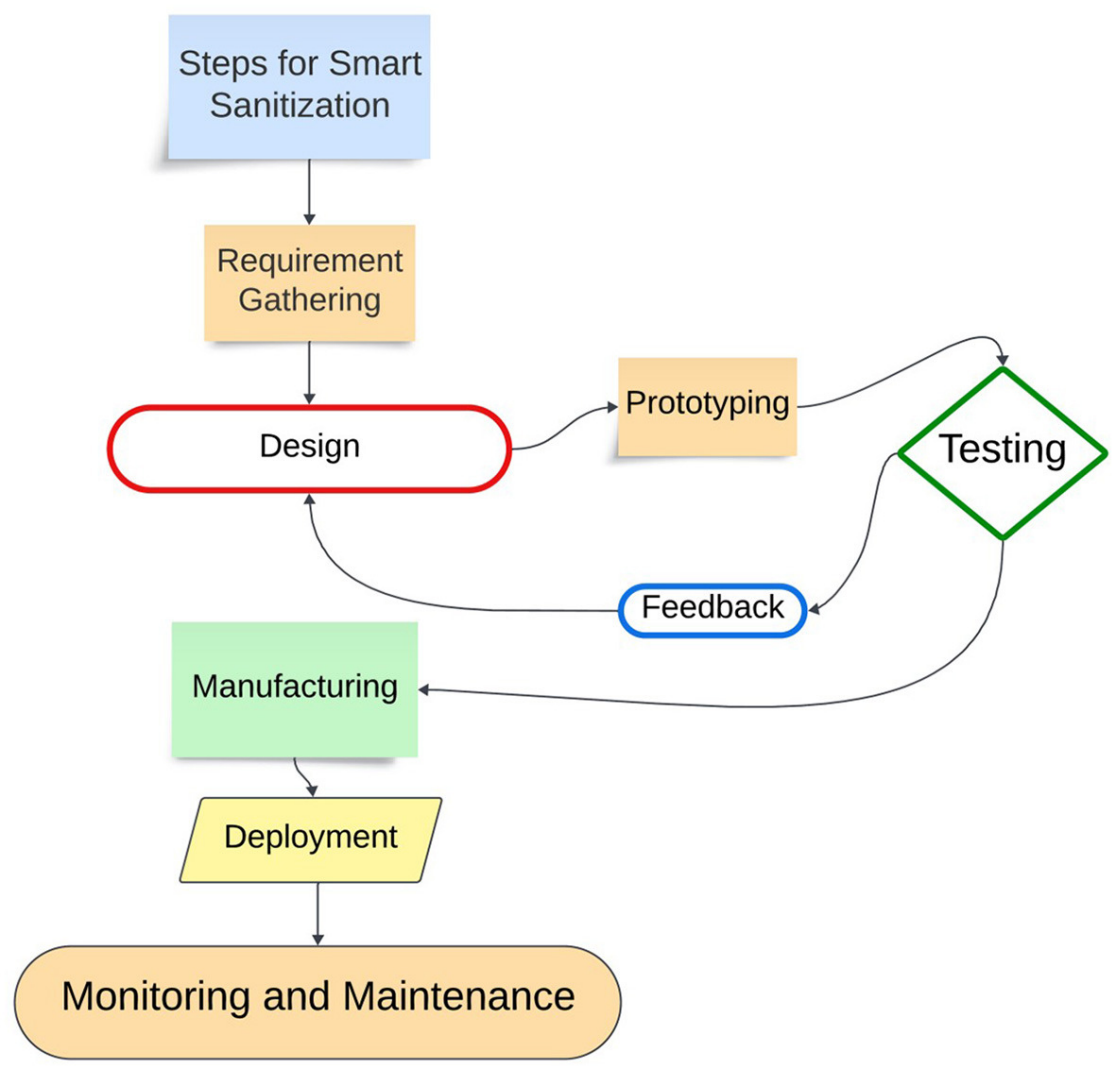
A flow chart of the phases of the project methodology.

As depicted in Figure 2.

1. Requirement gathering:

- Initial step involves collecting requirements from stakeholders, including public transportation providers such as buses and metro operators. This ensures that the device aligns with user needs.

2. Design:

- Utilizing Computer-Aided Design (CAD) software, the device is designed comprehensively. This includes assessing its functionality, dimensional accuracy, and user-friendliness.

3. Prototyping:

- Following the design phase, a 3D-printed prototype of the device is constructed to validate real-time developments and facilitate testing and necessary adjustments.

4. Testing and feedback:

- Thorough testing of the prototype on bus handrails is vital, encompassing the assessment of its sanitizer application efficiency, user acceptance and durability. During Testing, the device will be used by cleaning staff in public transport and by the passengers in the public transports such as Buses, Metros, and Trains. The user interface of the device can be assessed to evaluate its ergonomic and intuitive design. Field Testing is crucial to substantiate the device's practical effectiveness and its suitability for real-world deployment. The parameters which can be accessed to understand the device' performance are time taken for fixing, time taken for initiating and completing the sanitization process, cleaning efficacy, time efficiency.

5. Manufacturing:

- After successful testing and approval, the device can be manufactured using cost-effective Additive Manufacturing processes, selecting appropriate materials.

6. Deployment:

- The manufactured devices are then deployed to their intended users, potentially on public vehicles, in healthcare facilities, schools, and commercial buildings.

7. Monitoring and maintenance:

- Continuous performance monitoring and regular maintenance of the deployed devices ensure ongoing effectiveness in sanitizing bus handrails. By adhering to this methodology, we can develop a handrail sanitizing device that is efficient, safe, and cost-effective.

### Comparative analysis on the materials

1. Tensile strength:

- High: Nylon Polyamide (PA).

- Medium to High: Acrylic (PMMA), Polyoxymethylene (POM), and Thermoplastic Polyurethane (TPU).

- Medium: Polypropylene (PP), Polystyrene (PS), and Thermoplastic Elastomer (TPE).

- Low to Medium: Acrylonitrile Butadiene Styrene (ABS), PC/ABS, PC/PBT, and PPE/PS.

- Low: Polyethylene (PE).

2. Flexibility:

- High: Acrylonitrile Butadiene Styrene (ABS), Nylon Polyamide (PA), Polyethylene (PE), Thermoplastic Elastomer (TPE), and Thermoplastic Polyurethane (TPU).

- Medium: Polypropylene (PP), Polystyrene (PS), and Polyoxymethylene (POM).

- Low: Acrylic (PMMA), PC/ABS, PC/PBT, and PPE/PS.

3. Impact strength:

- High: Acrylonitrile Butadiene Styrene (ABS), Nylon Polyamide (PA), Polyethylene (PE), Polystyrene (PS), PC/ABS, PC/PBT, and PPE/PS.

- Medium to High: Acrylic (PMMA), Polyoxymethylene (POM), and Thermoplastic Elastomer (TPE).

- No Information: Polycarbonate (PC) and Thermoplastic Polyurethane (TPU).

4. Electrical insulation:

- Yes: Nylon Polyamide (PA), Polyethylene (PE), Polyoxymethylene (POM), Polypropylene (PP), Thermoplastic Polyurethane (TPU), and PC/PBT.

- No: Acrylic (PMMA), Acrylonitrile Butadiene Styrene (ABS), Polycarbonate (PC), Polystyrene (PS), Thermoplastic Elastomer (TPE), PC/ABS, and PPE/PS.

5. Temperature resistance:

- High: Acrylic (PMMA), Nylon Polyamide (PA), Polycarbonate (PC), Polyoxymethylene (POM), Thermoplastic Polyurethane (TPU), and PC/PBT.

- Medium: Acrylonitrile Butadiene Styrene (ABS), Polypropylene (PP), and PC/ABS, PPE/PS.

- Low: Polyethylene (PE), Polystyrene (PS), and Thermoplastic Elastomer (TPE).

6. Chemical resistance:

- Strong: Acrylic (PMMA), Nylon Polyamide (PA), Polyethylene (PE), Polyoxymethylene (POM), Polypropylene (PP), and Thermoplastic Elastomer (TPE).

- Medium: Acrylonitrile Butadiene Styrene (ABS), and PC/ABS, PPE/PS.

- Weak: Polycarbonate (PC).

- No Information: Polystyrene (PS) and Thermoplastic Polyurethane (TPU).

7. Cost (low to high):

- Low: Polypropylene (PP), Polystyrene (PS), Polyethylene (PE), and PC/ABS, PPE/PS.

- Medium: Acrylic (PMMA), Acrylonitrile Butadiene Styrene (ABS), Polypropylene (PP), PC/ABS, and PPE/PS.

- High: Nylon Polyamide (PA), Polycarbonate (PC), Polyoxymethylene (POM), Thermoplastic Elastomer (TPE), Thermoplastic Polyurethane (TPU), and PC/PBT.

8. Water resistant:

- Yes: All materials like Acrylic (PMMA), Acrylonitrile Butadiene Styrene (ABS), Nylon Polyamide (PA), Polycarbonate (PC), Polyethylene (PE), Polyoxymethylene (POM), Polypropylene (PP), Polystyrene (PS), Thermoplastic Elastomer (TPE), Thermoplastic Polyurethane (TPU), PC/ABS, and PPE/PS are water resistant.

9. Recyclability:

- High- Acrylic Acrylic (PMMA), Acrylonitrile Butadiene Styrene (ABS), Nylon Polyamide (PA), Polycarbonate (PC), Polyethylene (PE), Thermoplastic Elastomer (TPE), Thermoplastic Polyurethane (TPU), and PC/ABS.

- Low- Polyoxymethylene (POM).

10. Environmental impact:

- Lesser Emissions- polypropylene (PP) and Polyethylene (PE).

- High Impact- Acrylic (PMMA), Acrylonitrile Butadiene Styrene (ABS), Nylon Polyamide (PA), Polycarbonate (PC), Polyoxymethylene (POM), Polystyrene (PS), Thermoplastic Elastomer (TPE), Thermoplastic Polyurethane (TPU), and PC/ABS.

These chemicals can cause pollution during production get into the water systems, impacting aquatic life and contaminate water bodies.

Inference:

Nylon Polyamide (PA) stands out for its high tensile strength, flexibility, impact strength, electrical insulation, temperature resistance, and chemical resistance, but it comes at a higher cost. Polyethylene (PE) is cost-effective, water-resistant, and offers high impact strength, but it has lower tensile strength. Polycarbonate (PC) provides high tensile strength and temperature resistance but has weak chemical resistance. Acrylic (PMMA) is temperature-resistant and chemically strong but lacks in impact strength and flexibility. Thermoplastic Polyurethane (TPU) is flexible and temperature-resistant with high tensile strength. Polypropylene (PP) can be considered for its high flexibility, Recyclability, fewer environmental impact, electrical insulation, and low cost. The choice depends on specific application requirements, balancing factors such as strength, flexibility, cost, and environmental conditions. Considering the need to develop a cost-effective sanitizing device capable of withstanding wear and tear during operation, Polypropylene emerges as a suitable material choice. Its durability and lower cost compared to other plastic materials make it ideal for producing device components.

## Manufacturing methods

In terms of manufacturing methods, various options exist, each with its own advantages and limitations:

Plastic extrusion: Ideal for creating continuous profiles with uniform cross-sections, such as tubes or pipes.

Injection molding: Preferred for mass-producing plastic products due to its high output rate and consistent quality. Suitable for a wide range of products including automotive parts, electronic components, medical devices, consumer plastics, and furniture parts.

Rotational molding: Suited for hollow products with complex shapes, such as tanks, containers, and playground equipment.

Injection blow molding: Particularly useful for producing hollow objects in large quantities, such as bottles and containers.

Vacuum casting: Best for creating prototypes or low-volume production of parts with fine details and complex geometries.

Thermoforming & vacuum forming: Suitable for producing large, shallow parts with uniform wall thickness, like trays, packaging, and housings.

Compression molding: Effective for producing large, fairly simple parts with low to moderate volume requirements, such as appliance parts and electrical components.

When selecting a manufacturing method, it's essential to consider factors such as product complexity, material requirements, volume/cost considerations, and lead time:

Product characteristics: Assess whether your product has complex internal features or tight tolerance requirements, which may influence the choice of manufacturing method.

Material: Consider the functional, aesthetic, and cost requirements of your product and select a material that aligns with these criteria.

Volume/cost: Evaluate the total volume of products required and choose a manufacturing process that balances initial tooling costs, per-part cost, and production volume.

Lead time: Determine how quickly parts need to be produced and select a manufacturing process that meets your timeline requirements, whether it's rapid prototyping or high-volume production.

Based on the need for a low-cost manufacturing approach to produce parts for the sanitizing device efficiently and quickly, injection molding may be the preferred method, given its high output rate and cost-effectiveness for mass production.

### Wheels and its types

Wheels are crucial for the mobility of devices or vehicles, providing the necessary friction for smooth movement. A device designed for tree climbing, for instance, utilizes cylindrical wheels to securely mount and grip the circular surface of the climbing rod (17). Similarly, Ladd et al. created a pole climbing robot with cylindrical wheels for efficient pole climbing (18). Sadeghi et al. introduced a pole climbing robot with curved wheels, enhancing surface grip, and implemented a spring mechanism for added tension (19). Xu et al.'s cable climbing robot utilizes curved wheels for seamless mobility along the cable (20). In designing a cost-effective device, the choice of wheels significantly impacts mobility, ensuring optimal friction between the surface and the contact point.

### Sensors for making a device smart

To transform a device into a smart one, the integration of sensors is pivotal for its operation. Smart devices leverage a variety of sensors to gather data either from their surroundings or the device itself, enabling them to respond intelligently to different scenarios. Below is a compilation of common sensor types employed in smart devices:

1. Accelerometer

- Measuring properties: Acceleration forces.

- Uses: Enables devices to detect motion, orientation, and vibration.

- Applications: Smartphones, fitness trackers, and gaming controllers.

2. Proximity sensor

- Measuring properties: Distance between objects.

- Uses: Detects the presence of nearby objects without physical contact.

- Applications: Collision avoidance, automated doors.

3. Temperature sensor

- Measuring properties: Temperature difference.

- Uses: Monitors environmental conditions and adjusts device operations accordingly.

- Applications: Climate control systems, wearable health monitors.

4. Capacitive sensor

- Measuring properties: Capacitance change due to pressure.

- Uses: Detects touch or proximity by measuring changes in capacitance.

- Applications: On/off buttons in devices, stress analysis in materials.

5. Pressure sensor/barometer

- Measuring properties: Atmospheric pressure.

- Uses: Enables devices to determine altitude and predict weather changes.

- Applications: Climate monitoring, altitude tracking.

6. Microphone/sound sensor

- Measuring properties: Sound frequency.

- Uses: Detects sound waves and measures sound intensity or frequency.

- Applications: Voice-controlled devices, noise level monitoring.

When these sensors are combined with appropriate processing and communication capabilities, devices gain the ability to collect, analyze, and respond to data intelligently, thus earning the designation of being “smart.”

### Device as a smart miniature robot

The smart sanitizing device, envisioned as a miniature robot, represents a cutting-edge solution to the pressing need for efficient and accessible sanitization. This compact robot, inspired by the principles of miniaturization and intelligent design, is tailored to navigate diverse environments seamlessly. Its miniature size allows it to maneuver through intricate spaces with ease, ensuring thorough sanitization in areas that might be challenging for traditional methods. Equipped with advanced sensors, the robot can detect and analyze surfaces, intelligently identifying high-touch areas and applying sanitization precisely where needed. The integration of a microcontroller facilitates automated movements and adaptive responses, enabling the miniature robot to operate autonomously. This innovative approach not only enhances the effectiveness of sanitization but also promotes efficiency and accessibility, making it a versatile solution for various settings.

In addition to its functional prowess, the miniature robot exhibits a sleek and unobtrusive design, minimizing its impact on the surroundings. Its compact form factor allows for easy deployment in crowded spaces, ensuring comprehensive sanitization without disrupting regular activities. The device is crafted from cost-effective and lightweight materials, aligning with the goal of creating an affordable yet highly efficient sanitization solution. As a miniature robot, it embodies the convergence of technology and practicality, offering a forward-looking response to the global demand for enhanced hygiene measures.

### Comparative analysis

1. Comprehensive Sanitization Mechanism:

° Our device employs a dual-action sanitization process that combines UV-C light and a disinfectant spray, ensuring thorough sanitization of surfaces.
° Advantage: Unlike many existing solutions that rely solely on one sanitization method (either UV-C or disinfectant spray), our device offers enhanced efficacy by utilizing both methods simultaneously.

2. Automation and efficiency:

° The device is designed for automated operation, reducing dependency on manual intervention for sanitization processes.
° Advantage: This automation minimizes human error and ensures consistent sanitization results, which may not be achievable with manually operated systems currently in use.

3. Adaptability and integration:

° Our device is specifically engineered for integration into existing public transport infrastructure, such as buses and trains, without significant modifications.
° Advantage: This adaptability facilitates seamless integration into operational schedules and minimizes downtime during implementation, contrasting with solutions that require extensive retrofitting or infrastructure changes.

4. User safety and comfort:

° Safety features include motion sensors and automated shut-off mechanisms to prevent UV-C exposure during operation when passengers or personnel are nearby.
° Advantage: Ensuring user safety is paramount, distinguishing our device from some existing solutions that may pose risks of UV-C exposure without adequate safety measures.

5. Cost-effectiveness and sustainability:

° The device is designed to optimize resource use, such as energy and disinfectant consumption, contributing to long-term cost-effectiveness.
° Advantage: Compared to traditional sanitization methods that may incur higher operational costs due to manual labor and consumable expenses, our solution offers potential savings over time through efficient resource utilization.

## Opinion

In the development of this innovative smart sanitizing device, a technological marvel is presented, offering a practical solution to a significant global challenge. This endeavor stands as a testament to the transformative power of human ingenuity when faced with adversity. Navigating the complexities of a pandemic-ridden world, the fusion of technology and public health emerges as a potent force for positive change.

The proposed device, with its emphasis on accessibility and sustainability, transcends its immediate application, becoming a symbol of resilience and adaptability in the face of unforeseen challenges. It is not merely a device but a beacon of hope, signaling a future where innovation converges with compassion to safeguard the wellbeing of communities worldwide.

Considering the materials used in this endeavor, the choice becomes crucial. The selected materials not only contribute to the device's efficiency but also underscore a commitment to creating a lasting impact. Each material embodies a balance of strength, flexibility, and cost-effectiveness, mirroring the resilience aimed to be instilled in the communities served. This smart sanitizing device is not just a technological achievement; it is a tangible representation of dedication to addressing pressing global issues with a blend of cutting-edge solutions and a compassionate, sustainable approach.

Given the imperative for a low-cost manufacturing approach to efficiently and swiftly produce parts for the sanitizing device, injection molding may be the preferred method. Its high output rate and cost-effectiveness make it well-suited for mass production.

The importance of wheels in facilitating the mobility of devices is evident, particularly in specialized applications such as tree climbing and pole traversal by robots. The choice of wheel design, whether cylindrical or curved, plays a critical role in ensuring effective surface grip and overall device performance. Various innovative solutions, such as incorporating springs for added tension or utilizing specific wheel shapes, demonstrate the evolving nature of technology in addressing unique challenges.

References to tree pruning robots, pole climbing robots, and cable climbing robots highlight the versatility of wheel applications in diverse scenarios. The emphasis on cost-effective design further underscores the practical considerations in developing these devices.

The in-depth exploration of wheel designs for mobility in climbing devices showcases the intersection of technology and engineering, contributing to the evolution of solutions for specific tasks. These advancements, as evidenced by the referenced projects, signify the ongoing commitment to developing efficient and adaptable devices for various practical applications.

The synergy of the sensors, coupled with appropriate processing and communication capabilities, empowers devices to collect, analyze, and respond to data intelligently, thereby earning the designation of being “smart.” The inference underscores the versatile applications of sensors in enhancing device functionality and responsiveness to various environmental stimuli.

The concept of a smart sanitizing device in the form of a miniature robot is a testament to the innovative spirit driving advancements in technology. This miniature robot not only addresses the critical need for effective sanitization but also introduces a novel approach by leveraging automation and intelligence. The ability of the device to autonomously navigate and sanitize various spaces, especially those with intricate layouts, marks a significant step forward in the realm of hygiene solutions. Its unobtrusive design and cost-effective construction make it a pragmatic choice for widespread deployment, potentially revolutionizing the way we approach sanitization in public spaces, homes, and beyond.

The device can be designed without electronic components which purely run on mechanical energy. As a result, the device requires minimal maintenance and generates no electronic waste throughout its operational lifespan. Thus it can become as a sustainable product to the environment. The device can be either designed with electronic to a fully automatic device or a semi automatic device with purely mechanical energy. The design of the handrail sanitizing robot will prioritize user-friendliness, ensuring that anyone can operate it with ease.

As we witness the integration of intelligent robotics into everyday solutions, the miniature smart sanitizing robot exemplifies the potential of technology to enhance public health measures. The seamless blending of robotics, sensors, and efficient programming underscores the adaptability of modern solutions to meet evolving challenges. The development and adoption of such innovative devices not only signify a commitment to health and safety but also pave the way for a smarter and more responsive approach to sanitation in our daily lives.

### Sustainability of the device

1. Durability and reliability:

° The device is constructed using high-grade materials such as stainless steel and reinforced polymers, which are known for their durability and resistance to wear and tear.
° Advanced construction techniques, including weatherproofing and shock absorption mechanisms, have been employed to ensure the device can withstand harsh environmental conditions and regular usage over extended periods.
° Extensive testing has been conducted to ensure the device's components, such as sensors and connectors, maintain their performance and accuracy throughout the device's lifecycle.

2. Maintenance protocols:

° A detailed maintenance schedule has been developed, which includes regular checks and servicing intervals to ensure optimal performance.
° Maintenance procedures involve routine inspections, cleaning of sensors and external parts, and firmware updates to address any potential issues.
° Solutions for common issues, such as sensor recalibration and part replacements, are provided in the user manual to facilitate easy maintenance by the transport system's technical staff.
° The device's modular design allows for quick and straightforward replacement of individual components, minimizing downtime and ensuring continuous operation.

3. Cost-effectiveness:

° An analysis of the maintenance costs has shown that they are significantly lower than the benefits provided by the device in terms of improved efficiency and user satisfaction in public transport systems.
° The device's robust design reduces the frequency and cost of repairs, contributing to long-term savings for transport operators.
° By improving the efficiency of public transport systems, the device helps increase ridership and revenue, further justifying the investment in its maintenance and upkeep.

4. Environmental impact:

° The design and manufacturing process of the device prioritize environmental sustainability. Recyclable materials are used wherever possible, and efforts are made to minimize waste during production.
° The device's energy-efficient operation helps reduce the overall energy consumption of the transport system, contributing to lower carbon emissions.
° End-of-life disposal protocols are established to ensure that the device and its components can be recycled or disposed of responsibly, minimizing their environmental footprint.

### User interactions

1. User interaction studies:

° We conducted several field studies involving a diverse group of public transport users to gather comprehensive feedback on the device's functionality and user experience.

2. Feedback analysis and adjustments:

° Based on the feedback, users appreciated the device's ease of use and intuitive interface. However, some users pointed out that the initial calibration process was slightly time-consuming.

- Adjustment: We streamlined the calibration process, reducing the setup time by incorporating automated calibration features that require minimal user intervention.

° Users also indicated that the device's display could be challenging to read under direct sunlight.

- Adjustment: We upgraded the display to a high-contrast, anti-glare screen, ensuring visibility under various lighting conditions.

° Feedback highlighted a desire for more detailed notifications regarding transport schedules and delays.

- Adjustment: We enhanced the notification system to provide real-time updates and added customizable alert settings to meet individual user preferences.

2. Impact of adjustments:

° The adjustments based on user feedback have significantly improved user satisfaction and device performance.

3. User-centric design philosophy:

° Our commitment to a user-centric design philosophy ensures that the device evolves in response to user needs and preferences.
° By prioritizing user feedback, we can continuously refine the device to enhance its usability and effectiveness in public transport systems.

## Conclusion

The handrail sanitizing device outlined is designed to meet specific attributes essential for its successful implementation. The efficacy of the device is ensured through the utilization of liquid sanitizer, a well-established and proven method for effectively eradicating germs from handrails. Importantly, the device prioritizes user-friendliness, featuring a straightforward installation process and easy operation to cater to individuals without technical expertise. This not only enhances safety during human interaction but also contributes to environmental friendliness when used correctly. Furthermore, the emphasis on affordability ensures that the device remains cost-effective, promoting accessibility to a broad range of users. The device has significant implications for public health and hygiene, offering a scalable solution that can be adapted and improved upon in future iterations. By integrating smart technologies or exploring sustainable materials, further advancements could enhance its effectiveness and reduce environmental impact. Altogether, these attributes underscore the device's potential as an effective, user-friendly, safe, and affordable solution for handrail sanitization in various settings.

## Author contributions

JP: Conceptualization, Funding acquisition, Investigation, Methodology, Project administration, Resources, Software, Writing – original draft, Writing – review & editing. DD: Resources, Visualization, Writing – review & editing. DJ: Formal analysis, Validation, Writing – review & editing. SL: Methodology, Validation, Writing – original draft.
